# Nature-based solutions for improving food security: A systematic global review

**DOI:** 10.1016/j.heliyon.2024.e36082

**Published:** 2024-08-14

**Authors:** Hoang Minh Nguyen, Huu Loc Ho, M.S. Babel, Natthachet Tangdamrongsub, Sushil Kumar Himanshu, Perrine Hamel, Edward Park

**Affiliations:** a Water Engineering and Management, Asian Institute of Technology, Thailand; b Earth Systems and Global Change Group, Wageningen University and Research, Wageningen, the Netherlands; c Agricultural Systems and Engineering, Asian Institute of Technology, Thailand; d Asian School of the Environment, Nanyang Technological University, Singapore; e National Institute of Education, Nanyang Technological University, Singapore; f Earth Observatory of Singapore, Nanyang Technological University, Singapore

**Keywords:** Nature-based solutions, Food security, Hidden hunger, Water security, Agriculture sustainability

## Abstract

Nature-based solutions (NBS) have been promoted as a holistic way to solve a variety of societal issues while benefiting biodiversity at the same time. To date, applications of NBS approaches that help ensure food security have yet been systematically reviewed. In this paper, we critically review the specific NBS for food security, highlighting their limitations, to provide recommendations that promote their applications for improving global food security. We accessed and evaluated publications on four different scholastic databases, and our systematic review of relevant materials indicated that many NBS approaches can be applied to enhance food security dimensions individually or together. However, there is a strong bias towards food availability, and not enough research has been done to link NBS with improvements in food access and utilization. Over 80 % of the reviewed papers were of short-term studies or without specific timeframes, and 25 % offered no information on the economic effectiveness of NBS. Environmental benefits of NBS were explicitly described in about 60 % of these papers, and biodiversity enhancement was measured in only about 10 %. We, therefore, recommend future applications of NBS to safeguard food security be shifted to food access and utilization with careful consultation with local communities to address their specific context, using indicators that are easily measured and managed. Systematic monitoring regimes and robust and diversified financial support systems are also equally important in efforts to successfully implement NBS. Moreover, environmental and societal benefits, especially water productivity and biodiversity, must be incorporated into the planning and design of NBS.

## Highlights

• Nature-based solutions can enhance food security dimensions together or separately.
• Much emphasis on volumes of food production; little focus on access and utilization.
• Lack of information on economic effectiveness and biodiversity benefits.
• Robust monitoring and evaluation regime, and financial supports are recommended.

## Graphical abstract

**Figure:**
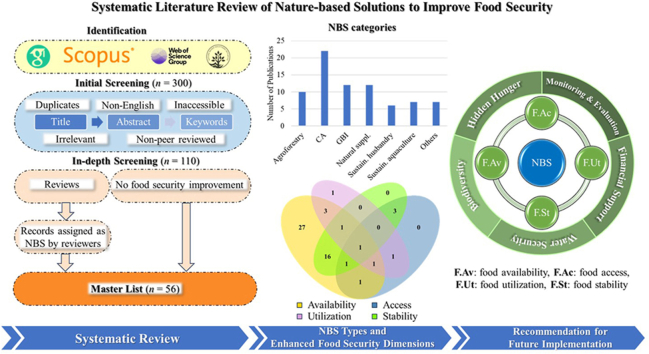

## 1 Introduction

Nature-based solutions (NBS) are defined by the International Union for the Conservation of Nature (IUCN) as “actions to protect, sustainably manage, and restore natural or modified ecosystems, which address societal challenges effectively and adaptively, simultaneously providing human well-being and biodiversity benefits” [1]. NBS involve sustainable planning, design, environmental management, and engineering practices that weave natural features or processes into the built environment to build more resilient communities. In other words, NBS are actions inspired by, supported by, or copied from nature, that deploy various natural features and processes, are resource efficient, and adapted to systems in diverse spatial areas, facing social, environmental, and economic challenges [2]. It is a recently coined concept that started to circulate in the scientific community in the early 2000s and was built on the concept of promoting a holistic and integrated approach to promote conservation, restoration, and sustainable management of ecosystems while also taking into account the increasing demands placed on ecosystem services [2,3]. It has been considered an ‘umbrella term’ that encompasses other ‘nature-based’ approaches such as Ecological Engineering (EE), Catchment Systems Engineering (CSE), Ecosystem Approach (EA), and Ecosystem-Based Adaptation (EBA) [4,5] (see Table 2 in Nesshöver et al. [4] for a concise overview of different concepts related to NBS). NBS has been employed by a wide range of stakeholders as a requirement to achieve sustainable development in responses to a variety of societal challenges such as human health [6], and climate change mitigation and adaptation [7].

Despite being a major global challenge and able to transcend all Sustainable Development Goals [8], food security has not been a major focus of research on the applications of NBS in comparison to other fields [[9], [10], [11]]. NBS applications to improve food security are often in conjunction with other types of interventions but have not been the main focus. Reviews of NBS in the context of sustainable agriculture and food systems often do not clearly identify how specific interventions can improve food security, especially under the pressure of an ever-growing global population and climate change impacts. For example, Simelton et al. [12] and Polo-Ballinas et al. [11] produced comprehensive reviews of NBS that focused on the categorization of functions, mechanisms, and scales of such practices and provided no specific information on what particular NBS practice may improve a specific food security dimension. Definitions of food security dimensions, namely availability, access, utilization and stability, are provided in Table 1. Another example is Mrunalini et al. [13] who reviewed the effectiveness of using NBS to improve soil health with only indirect implications on food production, quality and sustainability.

**Table 1:** Description of food security dimensions, based on the definition of food security by the Food and Agriculture Organization of the United Nations (FAO) [14]).

Food security dimension	Description
Availability	Availability is achieved if adequate food is ready to have at people's disposal
Access	Access is ensured when all households and all individuals within those households have sufficient resources to obtain appropriate foods for a nutritious diet.
Utilization	Food utilization reflects the quantity and quality of dietary intake and the nutritional and health status of the people.
Stability	Food insecurity is ultimately determined by the stability of the above three dimensions over time, that is, everyone, at the population, community, household and individual levels, must have sustained access to adequate and nutritious food at all times.

**Table 2:** Descriptions of improvements to food security dimensions, adapted from the Food and Agriculture Organization of the United Nations (FAO) [14], in comparisons to conventional methods.

Food security dimension	Description of improvement
Availability	Increases in food crop, livestock, aquaculture production
Access	Increases in the number of people (or the proportion of population) with access to food
Utilization	Increases in the nutrition quality of the food products produced
Stability	Increases in or maintenance of the production or quality of food products over time

Recent efforts to review NBS and food security, e.g. Refs. [9,10,15,16], are limited in scope to urban environments and specific aspects of food security. There is little focus on the synthesis of global scientific knowledge on NBS and food security in rural agricultural contexts. Past efforts to increase food security have largely focused on agricultural and ecosystem management practices that heavily relied on extensive use of chemical fertilizers and pesticides, and water from irrigation systems [17,18]. Consequently, the challenge to sustain and increase the benefits of agricultural production while preserving the quality and quantity of ecosystem services provided by the Earth's land and water resources remains [11,19,20].

Moreover, in spite of the effort of IUCN to codify an overarching definition, the meaning of NBS is not yet uniform and depending on the use of a particular term, the aims and targeted strategies of NBS projects can vary significantly [3,4]. While the wide variability of NBS framing can facilitate discussion and innovation, and enable applications in multiple different fields, the lack of a comprehensive formulation can lead to miscommunication and confusion, and complicate the upscaling and transferability of a project's findings [4]. A comprehensive review of NBS approaches explicitly targeting food security is therefore essential in establishing the framework in which their benefits in relation to specific food security dimensions and geographic regions are clearly identified. More importantly, one needs to find NBS approaches that address food security and are applicable to the Global South, an often-neglected region in studies on NBS effectiveness [5,7,12].

The overall goal of this review is to identify and synthesize the current status and development of NBS that specifically address food-security-related issues in non-urban settings. The objectives of the review are 1) to identify evidence of NBS around the world that successfully improve one or multiple food security dimensions in rural environment; 2) to identify the limitations to the implementation of these NBS; and 3) to identify the enabling factors that help promote the application of NBS on improving global food security.

## 2 Methods

### 2.1 Literature search and screening processes

**Fig. 1:**
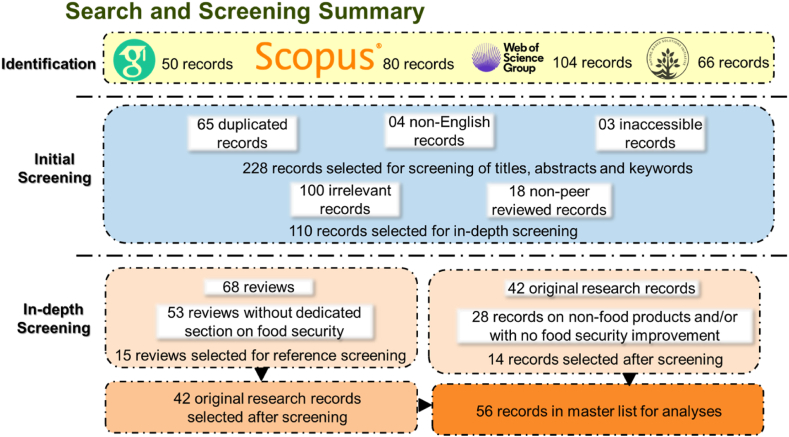
PRISMA statement process for the selection of articles for the database of this review.

We followed the Preferred reporting items for systematic review and meta-analysis protocols (PRISMA-P) 2015 statement [21] to establish the literature database. The literature search involved two main phases: 1) Identification of potential publications; and 2) Screening the identified publications to detect suitable ones for analyses and to remove ineligible ones (Fig. 1).

For the first phase, we searched four different databases, namely Google Scholar, Scopus, Web of Science and Nature-based Solution Initiative using the query terms [Nature-based solutions] AND [food security], and [NBS] AND [food security], excluding papers not in the English language and limited to ones published by December 31st, 2022. The queries resulted in 300 publications that were included in the screening database.

In the initial screening process, we excluded publications that were duplicates of others in the database (n = 65), those that were not in the English language (n = 4) and ones in which access to full text was not available (n = 3). We then scanned the titles, abstracts and keywords of the resultant 228 publications to eliminate sources not related to nature-based solutions and food security in agricultural or rural contexts (n = 100), and then records that did not meet peer-reviewed standards such as posters, policy briefs, editorials and book chapters (n = 18). In total, at this stage, 190 publications were removed.

In order to identify and quantify the efficiency of an NBS approach in enhancing food security, further screening was required to only include publications with empirical results for detailed analysis. We filtered out sources that were not empirical studies (n = 68) and performed in-depth screening by scanning the abstract, introduction and results of the remaining ones to identify references in which the research subjects were food products (as defined by Annexes 3, 5 and 6 in Food and Agriculture Organization of the United Nations (FAO) [22]) in which there was evidence of improvements in at least one food security dimension (Table 2). After screening, only fourteen articles remained, seven in which the authors self-identified that they applied NBS approaches and seven in which the practices were identified as food security related NBS by the Nature-based Solution Initiative database [23]. To build a more robust database, we screened the filtered reviews and identified those with specific sections on food security (n = 15). Citations in those sections were screened to find additional sources in which the practices were labeled as NBS by the reviewers. Additional 390 articles were found to be relevant and subjected to in-depth screening which resulted in another 42 sources being added to the database.

### 2.2 Analysis

#### 2.2.1 Preliminary analysis

We produced descriptive statistics of the selected sources (n = 56) including the year in which the study was published, the continental distribution of the study areas, and the food products they targeted. Those with study areas located on different continents are labeled transcontinental. The locations of study areas were further classified into Global North and Global South, as defined by Castro Torres et al. [24]. The studied food products were grouped according to classifications by FAO [24].

#### 2.2.2 In-depth analysis

To better analyze the efficiency of the NBS interventions in addressing food security, we categorized them by the food dimensions they explicitly improved; the type(s) of the NBS approach(es) described in the publications; the length of time that the study covered; whether the authors conducted any cost and benefit analyses; and if the interventions produced any other environmental benefits. To correspond an NBS approach with a specific food security dimension, we relied on the authors’ claims, i.e., using the terms availability, stability, utilization, access and their synonyms, respectively. If these terms were not presented, we analyzed the Results sections to ascertain the dimension that had been improved (see Table 2 for details on descriptions of improvement). The study period of each study was characterized according to Raymond et al. [25] which proposed the three broad temporal scales of short (within 5 years), medium (5–10 years) and long-term (over 10 years).

With regards to the type of NBS approaches, after in-depth screening, seven overall categories emerged from the previously used classification, 1) Agroforestry [26]; 2) Conservation agriculture (CA) [27]; 3) Blue/green infrastructure (BGI), engineering solutions that physically regulate water and soil [12]; 4) Natural supplementation, in the forms of soil amendment [28] and agronomic biofortification [29]; 5) Sustainable livestock husbandry [30], 6) Sustainable aquaculture [31]; and 7) Others.

### 2.3 Limitations of the study

This study did not take into account concepts that were often associated with NBS such as Ecosystem Approach (EA) [32] and Ecosystem-based Adaptation (EbA) [33]. Although the selection of keywords employed in our design drastically reduced the number of eligible publications in our search, it helped identify the trend of developments that were explicitly classified as NBS and resulted in improvements on specific food security dimensions while keeping the review manageable. In addition, as we would like to focus on the development of NBS applications among the scientific community, we opted to remove government documents and policy briefs from our database and only considered peer-reviewed publications. As a result, our study did not analyze government action plans and technical reports that involved both food security and biodiversity measures.

## 3 Results

### 3.1 Temporal and spatial distribution of research, and types of food products targeted

**Fig. 2:**
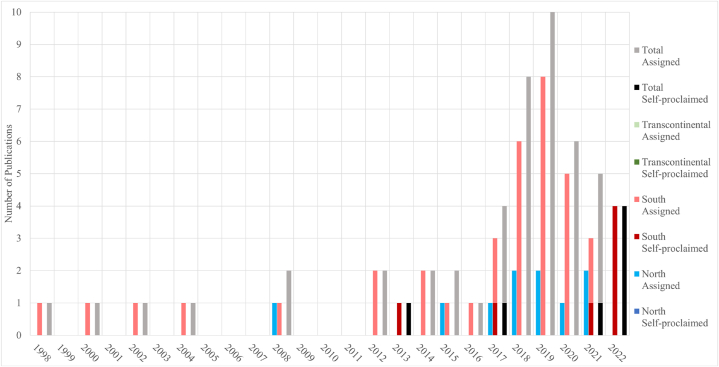
Temporal distribution of NBS research targeting food security.

**Fig. 3:**
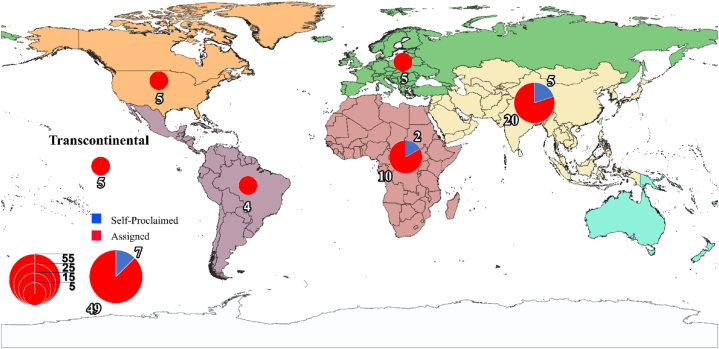
Geographical distribution of NBS research targeting food security.

**Fig. 4:**
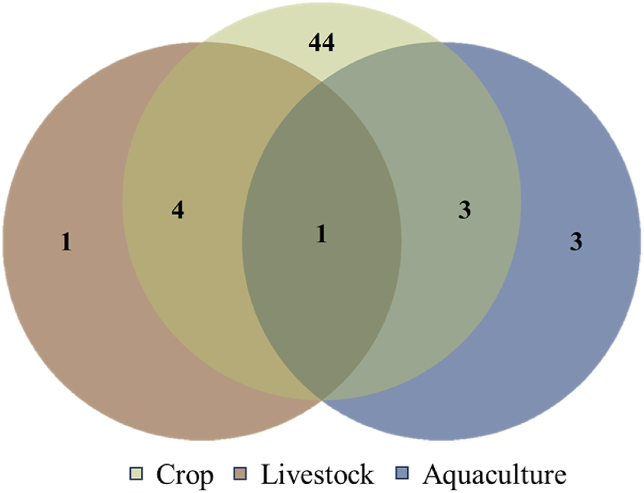
Targeted food product types of NBS research targeting food security.

Overall, the number of publications on practices identified as NBS approaches has increased consistently over time and most of the eligible publications identified in this paper were published after 2012 (Fig. 2). Notably, the number of self-proclaimed NBS studies, while small, has increased significantly since the early 2010s but none has come from the Global North. The majority of eligible sources (*n* = 49) were not initially labeled as NBS but assigned by reviewers at a later stage. In contrast to findings on reviews of NBS in urban settings, we found that most of the qualified articles in our database were on studies conducted in the Global South (*n =* 41) while a small number (*n* = 5) contained results from more than one specific continent (Fig. 3). The targeted food products in the reviewed articles significantly skewed towards crop products with the majority of studies (*n* = 44) being on crops only (Fig. 4). Studies that incorporate multiple different food types were uncommon (*n* = 8).

### 3.2 Food security dimensions targeted

**Fig. 5:**
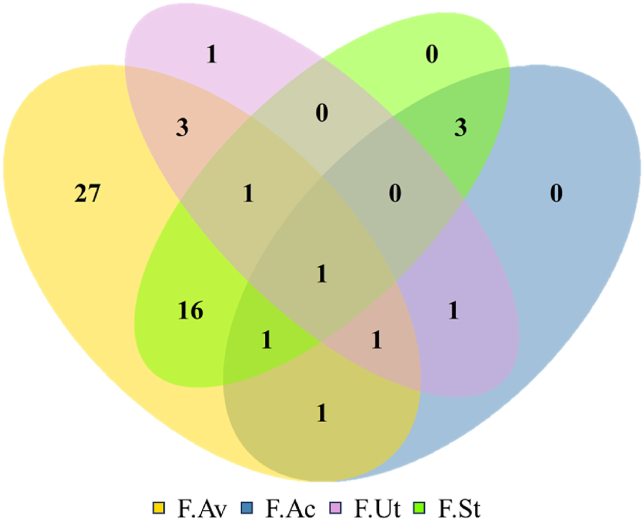
Relationships between the analyzed sources in this study, based on the food security dimension they enhanced. *Abbrev.*: F.Av: food availability, F.Ac: food access, F.Ut: food utilization, and F.St: food stability.

Our results showed that applications of NBS could improve food security in all of its dimensions. However, they also indicated a significant bias towards food availability and a lack of focus on other dimensions. Of the analyzed articles in this paper, 28 were on studies that targeted only one food security dimension and of which, 27 reported improvements on only food availability (Fig. 5) (see Table S1 in the Supplementary Material for more details). Among the studies addressing multiple dimensions simultaneously, 24 found enhancements on two dimensions simultaneously and among these, the most popular pairing was with availability and stability (*n* = 16). Reports on improving more than two dimensions at the same time were rare, with such outcomes being achieved in only four studies.

### 3.3 NBS approaches applied

**Fig. 6:**
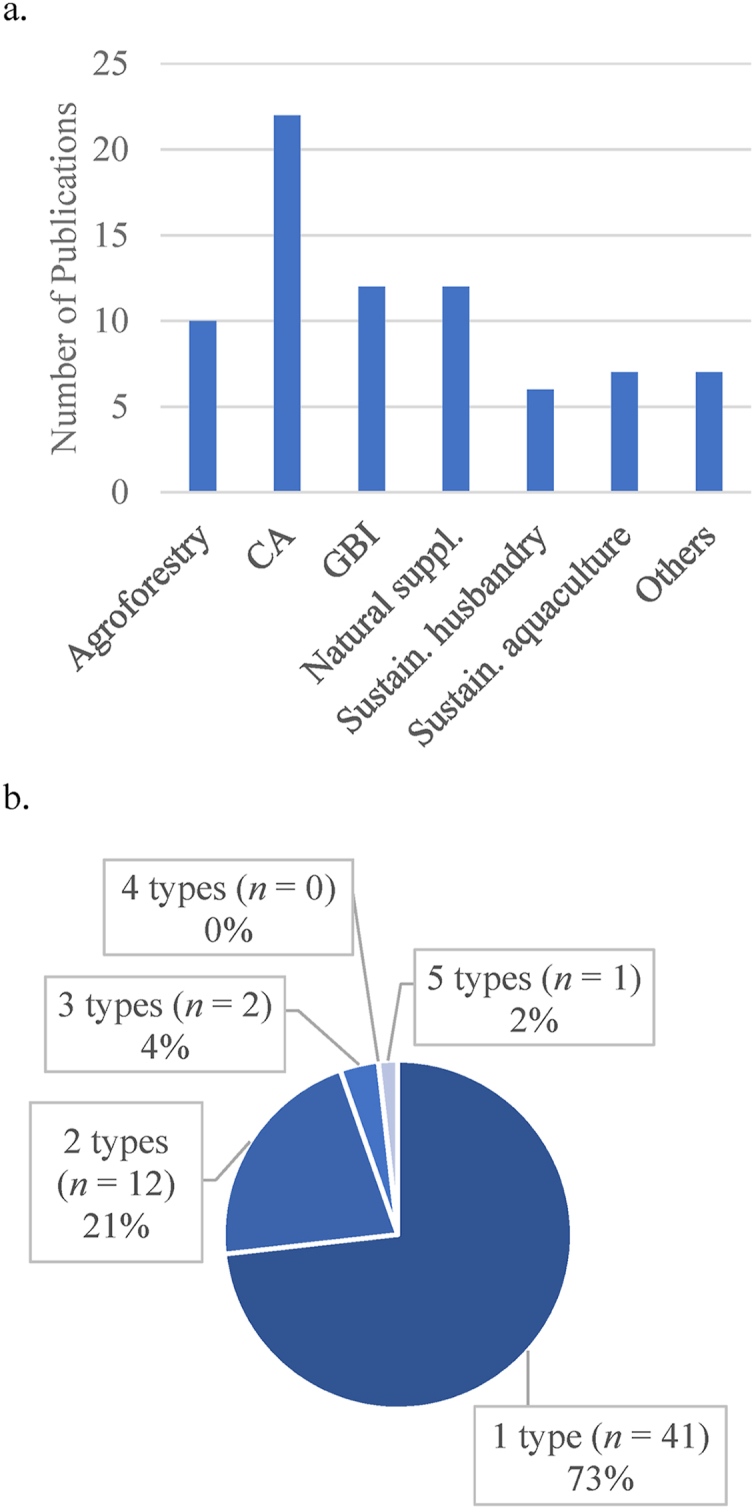
The categories of NBS applied in research targeting food security (a) and the number of NBS types applied in each study (b).

Multiple NBS approaches have been applied to enhance food security in rural agricultural environments (Fig. 6a). Most of the studies applied only one type of intervention and combinations of more than two NBS categories were rare (*n* = 3) (Fig. 6b.). CA, e.g., reduced/minimal/zero tillage, crop residue preservation and crop diversification, was the most popular category with multiple combinations of its components reported (*n* = 22). The number of studies that applied this type of intervention was nearly double that of studies with BGI and natural supplementations (*n* = 12 each).

### 3.4 Addressing food availability

Among the studies that found positive results on food availability, the applications of NBS skewed towards the production of cereal crops (Table 3 and Table S1 in the Supplementary material). Reports on improvements in the productions of other types of food products were rare and mostly came from studies that combined crop production with that of aquaculture or livestock (*n* = 7).

**Table 3:** Sumamry of characteristics of the reviewed publications on NBS targeting food security. See Tables S1-3s for detailed characteristics of individual publications.

	Food security dimension			
	Availability	Stability	Utilization	Access
Food product				
Crop only	42	16	6	2
Aquaculture only	2	1	0	1
Livestock only	1	1	0	0
Combination of two products	5	3	1	4
Combination of three products	1	1	1	1
Type of NBS applied				
Agroforestry only	3	2	0	1
Conservation agriculture only	15	5	5	1
Blue/green infrastructure only	4	4	0	0
Natural supplementation only	11	2	1	0
Sustainable husbandry only	1	1	0	0
Sustainable aquaculture only	1	0	0	0
Others	4	1	0	0
Combination of two NBS	10	4	1	4
Combination of three NBS	1	2	0	1
Combination of four NBS	0	0	0	0
Combination of five NBS	1	1	1	1
Duration				
Short-term (≤5 years)	43	18	8	4
Mid-term (5 to ≤10 years)	1	0	0	1
Long-term (>10 years)	4	2	0	2
Short-term x Mid-term^a^	2	1	0	0
Short-term x Long-term^a^	1	1	0	1
Mid-term x Long-term^a^	0	0	0	0
Economic evaluation				
No economic assessments	40	15	6	1
Investment assessment only	3	1	0	1
Benefit assessment only	5	5	1	5
Both assessments	3	1	1	1
Benefits beyond food security				
No benefits beyond food security	21	5	5	3
Water benefits	5	2	0	1
Biodiversity benefits	3	2	1	1
Benefits not stated above	15	10	2	3
Two benefits beyond food security	7	3	0	0
Three or more benefits beyond food security	0	0	0	0

a These papers reported results from multiple study areas with different study durations.

A diverse variety of NBS approaches were applied to enhance food availability as reported in our reviewed articles (Table 3 and Table S2 in the Supplementary Material). The most common approaches were CA (*n =* 20), specifically intercropping and/or crop rotation with cereal crops and legumes. In addition, some researchers found positive results when using nitrogen-fixing trees like *Faidherbia albida* which provided similar effects to legumes [34,35]. Applications of organic fertilizers such as livestock manure and sediment trap silt were the second most popular approach (n = 12). These types of fertilizers may be further fortified with or without organic compounds like algal extracts [36] or biodynamic compounds [37]. Positive results may also come from the conservative application of conventional chemical fertilizers enhanced with plant growth-promoting bacteria (PGPB) [38,39]. Among studies with BGI (n = 11), most utilized organic structures built by living vegetation or repurposed agricultural wastes. Examples included grass strips [40,41], windbreaks [42], shellfish reefs [43], and organic geotextiles [44,45]. Agroforestry applications were often incorporated with other approaches like sustainable livestock husbandry, BGI, and CA [42,46,47].

Publications on long-term studies were scarce as more than half of the relevant papers in this sub-section were on experiments conducted over only one year or one crop cycle, or offered no information on lengths of study periods (Table 3 and Table S3 in the Supplementary material). Among those with multi-year (or multi-cycle) records, almost all were of short-term to medium-term (≤10 years). The only long-term studies were on sustainable water utilization practice (∼30 years) [48] and zero-tillage agriculture (17 years) [49], categorized as BGI and CA approaches respectively.

Information on financial investment and/or benefits of the NBS practices applied was often not reported with only approximately 20 % of the reviewed papers in this sub-section (n = 11) providing this type of data (Table 3). Among these studies, many offered data on either net benefit or gross income resulting from applications of NBS but there were no uniform units. Reported values varied from aggregated income [34,50], to household income [46], and net return per hectare [51]. Few presented specific amounts of investment required to implement targeted NBS [51,52]. Some only reported benefits in the form of increased benefit-cost-ratios [52] or percentages of incomes in comparison to conventional agriculture [53].

Many of the publications (n = 30) reported benefits beyond direct improvement of agricultural production such as increases in carbon sequestration, nutrient storage, water storage, and biodiversity (Table 3 and Table S3). Heightened amounts of carbon stored underground in these studies were reported in organisms’ bodies, soil microbes, and total soil organic carbon, e.g., Refs. [37,43,54,55]. Applications of NBS to improve food availability were also found to result in higher levels of all essential nutrients, i.e., nitrogen, phosphorus, and potassium, and others in soil [36,56,57]. Other notable benefits included increases in water storage thanks to higher soil water retention [40,56,58] or higher water conservation by artificial means such as water refuges [51] and underground water capture [48]. Additionally, NBS could also reduce the water demand for crops and livestock, especially for thirsty crops like rice and soybean [47,51,54]. Although improvement in biodiversity might be inferred from practices that involved more than two species, for this analysis, only studies in which biodiversity was an explicit outcome were considered (n = 5). Their results indicated that food production could be enhanced at the same time as biodiversity at all levels, from genetics [53] to ecosystem functions [46].

### 3.5 Addressing food stability

Studies on NBS application and food stability were much more uncommon with less than half of the publications in our database identifying improvements in food stability (*n* = 22) and of which, 16 focusing solely on crop production (Table 3 and Table S1). Research on increasing protein production stability was almost always in conjunction with that of crops [[59], [60], [61]]. Exceptions were on silvofishery, i.e. aquaculture and mangrove forestry integration [62], and sustainable cattle husbandry [63].

Similar to sub-section 3.4, a diverse collection of NBS approaches was found to enhance food stability around the world and 30 % of them applied more than one approach (Table 3 and Table S2). About one third of the studies relevant to this dimension (*n* = 8) applied CA practices such as intercropping and crop rotation. Specifically, grain crops were always intercropped with legumes, e.g., pea, and common bean [[64], [65], [66]] but in crop rotation, legumes can be replaced by other grain crops [67]. In contrast to BGI applications to increase food availability, those used to raise food stability mostly were engineering practices such as mounds and dykes [46], and artificial water control [48,59,68]. Another distinction was that agroforestry was not combined with other practices to achieve the desired outcomes. With respect to food stability, numerous types of trees such as mangroves [62], nitrogen fixing trees [34,61] and palms [46] were able to support and maintain food production over time if grown together with crops.

Although all of these studies claimed to increase the resilience or sustainability of the food production system, many of them simply provided information on how production had been stabilized or how system resilience had been increased without presenting specific timeframes or long-term monitoring records to support their claims (Table 3 and Table S3). For instance, Garrett et al. [69] stated that selective crop breeding with resistance genes could increase crop resistance to pests with regard to projected climate change and related impacts and consequently, maintain food security but they provided no specific timeframes. Thorlakson et al. [61] and Wang et al. [63] employed social surveys to detect how farmers perceived the agricultural productivity in their land had changed over time without explicit timelines. Among those that provided data on year-to-year changes, eleven were of short-to medium-term only, and three were with long-term records but offered no *in-situ* biological data [48,59,62].

One observation shared between this sub-section and that on food availability is the absence of economic evaluation in the reviewed sources (Table 3 and Table S3). Only one third (*n* = 7) of the relevant sources contained any information on the financial aspects of the NBS approaches in question. Five of them described how farmers’ income increased in comparison to conventional agriculture in different units [34,46,59,60,63] while Rahman et al. [62] projected benefit-cost-ratio of different silvofishery systems in Bangladesh for a variety of future periods. None provided data on the investment cost of the respective NBS practices they applied except Thorlakson et al. [61] who reported a one-year investment support of ∼US$300/household in the form of supplies.

Benefits beyond food security were also frequently reported in the relevant resources. (Table 3 and Table S3). The results of over three quarters of these papers (n = 17) suggested that NBS could improve ecosystem health at the same time as food stability. Multiple of them reported increases in soil organic carbon while Kumar et al. [70] found rises in both soil and biomass carbon levels. Additionally, efforts to increase food stability could result in increases in nitrogen and phosphorus retention in soil [41,55,57,67] as well as decreases in soil loss [40,61,71]. Unexpectedly, changes to water storage and demand were only described in three publications with all involving BGIs [40,48,59]. Similarly, biodiversity was only explicitly measured in four studies, each of which focused on a different group of organisms [46,55,57,72].

### 3.6 Addressing food utilization

There was a significant difference between the number of studies with improvements on the dimensions of availability and stability, and those on the dimension of utilization. We could only identify eight publications in which positive outcomes on food stability were reported (Table 3 and Table S1). All of them were on studies that had crops as research subjects with the most common one being maize (n = 3) and wheat (n = 2). The only articles on protein production were on joined aquacultural and crop production in reforested areas [46] and integrated prawn–fish–rice farming [50] in Bangladesh.

While all types of NBS approaches identified in section 3.3 could increase food utilization, only CA practices were found to be effective in multiple different studies (Table 3 and Tables S1 and S2). Within this category, intercropping and crop rotation systems with grain crops and legumes were the most popular methods (n = 3) [64,[73], [74], [75]]. Interestingly, N'Dayegamiye et al. [73] reported that legumes in monoculture and in polyculture systems with a different grain crop can improve the yield and quality of maize and wheat monoculture in the subsequent season. Biofertilizer treatments of cow manure and microalgae in equal measures were the only example of natural supplementation in this sub-section [36].

None of the reviewed articles in this sub-section were from studies in which data were collected in more than one year or one crop season with the exception of Głowacka et al. [64]. They reported significant and stable increases in macronutrients (phosphorus, calcium and magnesium) in maize grain in intercropping with common bean and spring barley over the course of three years. In their design, maize quality was also found to be at higher levels when grown next to common bean than next to barley.

Data on economic outcomes was severely lacking in articles relevant to the food utilization dimension (Table 3 and Table S3). No articles offered detailed information on the financial investment required by their respective approaches. One mentioned an initial support over the course of 6 months but provided no exact values [46]. Financial gains were reported in two papers [46,50] but that of Ahmed et al. [50] was of hypothetical values up to ∼US$9.5 billion when different proportions of total seasonal rice fields were converted to prawn-fish-rice farming.

Additional benefits were not commonly reported in articles on NBS that enhanced utilization (Table 3 and Table S3). Only three of the eight articles described environmental benefits, specifically increases in biodiversity, extreme weather protection, and better soil nutrient storage. In reforested areas in Bangladesh, the combination of mangroves, fruit trees, fish, and ducks not only raised the species diversity within the community but also protected it from extreme weather events [46]. On the other hand, Brilhante et al. [76] suggested that crop diversification increased Cabo Verde's resilience in response to the adverse effects of climate change on the country's agricultural production. In addition, there was a significant rise in soil macro- and micro-nutrients contents in Dineshkumar et al. [36].

### 3.7 Addressing food access

There was the same number of publications on NBS that enhanced food access as that on food utilization (*n =* 8, Table 3 and Table S1). The research subjects were diverse with maize and common beans being the only food crops mentioned in different studies [34,47,76]. Interestingly and distinctly, the majority of them included protein productions (*n* = 6) and five of these were in conjunction with crop productions. The only exception was the silvofishery application in which shrimps and crabs were raised together in mangrove forests [62].

NBS practices with positive effects on food access were notably less diverse than those on other food security dimensions. For this specific section, we could identify no records on the effects of natural supplementation on food access (Table 3 and Table S2). Six were on studies that applied agroforestry practices, most commonly in the forms of alley cropping [34,47,60] and mangrove forest integration [46,62]. The only paper on conservation agricultural practices promoted the cultivation of diverse native legume crops in Cabo Verde archipelago [76]. Sustainable aquaculture practice of integrated prawn–fish–rice farming was also found to increase food access among poor rural communities in southwest Bangladesh [50].

Among the records analyzed for this sub-section, the proportion of those that reported extended study periods was significantly higher than those of other food security dimensions (Table 3 and Table S3). Three of them were on studies that utilized population surveys to find positive results in food access when applied agroforestry in southern Africa, Brazil, and China over extended periods (up to 20 years) [34,47,59]. Using a different approach, Rahman et al. [62] applied quantitative analyses to project the financial and economic outputs for different time horizons, from three to 35 years.

Another similarity was the ratio of papers in this category with information on economic assessments of the applied NBS which was significantly higher than those in any other dimensions. Three quarters of the reviewed pertinent sources (*n* = 6) indicated positive financial benefits but only four of them were with *in-situ* data. Monetary outputs reported by Ahmed et al. [50] and Rahman et al. [62] were only projections under different hypothetical scenarios. Moreover, while an initial 6-month support was cited in Ahammad et al. [46], there were no records on specific costs of implementing the respective NBS approaches in any papers.

Additionally, positive environmental outcomes were much more commonly found in this sub-section (Table 3 and Table S3). Two of them described significant reductions in resource demands, including land, water, and nutrients, when applying agroforestry practices in comparison with conventional approaches [47,59]. Additionally, studies with mangrove forest settings showed enhanced protection against extreme weather events [46,62]. Similarly, the switch from crops that are common but sensitive to climate change effects like maize was reported to improve food access for Cabo Verde's population [76]. Other benefits were increases in soil organic carbons [47] and in species diversity [46].

## 4 Discussion

### 4.1 Significance of the study

To the best of our knowledge, our study is the first one to systematically review publications that specifically describe the positive outcomes of NBS applications on food security dimensions in rural contexts. Additionally, the authors methodically categorized these practices according to targeted food products, types of applications, study durations, and economic assessment (see Tables S1-3s for more details) to assist fellow researchers with subsequent studies in the same field.

Based on the presented review, NBS approaches were found 1) to have positive impacts on one or more food security dimensions, 2) to be applicable to the production and quality of multiple food products, 3) to produce economic benefits, and 4) to concurrently enhance food security, water security and biodiversity. Nonetheless, we strongly stress that these benefits were not uniformly found across all reviewed publications due to a number of limitations. The most common ones were the narrow focus on food availability and crops, and the absence of long-term monitoring systems for most of the reviewed studies. Other significant constraints that hindered the holistic applications of NBS and limited their utilizations included the lack of economic evaluations of the NBS applied, and little focus on water and biodiversity benefits.

### 4.2 Food security dimensions targeted

Over 90 % of the publications in our database (51/56) produced positive outcomes in regard to food availability and 27 of these presented no improvements on any other food security dimensions (Table S1). These results were not unexpected as food availability is the prerequisite for other food security dimensions, and concerns on food security have predominantly concentrated on (and often conflated with) food availability [[77], [78], [79]]. At the same time, in 42 of them, the only research subjects were cereal crops. This narrow focus on the growth of crop production signifies a historical focus on a cereal-based diet with low intakes of proteins and micronutrients [80,81]. This type of nutrient deficiency is described as ‘hidden hunger’ and is particularly prevalent in low- and middle-income countries where poverty prevents a significant proportion of the population from accessing and utilizing nutritious foods [77,82]. Furthermore, the cumulative impacts of fast-growing populations, significant and negative effects of climate change, and rising demands for proteins and fat in people's daily diet can lead to an increasing number of people being consistently subjected to weak food security [83]. Therefore, it has been suggested that emphases on food security dimensions beyond availability would be the most important assessment of food security in order to develop appropriate interventions [78,79].

### 4.3 Timeframes of research implementation

Nearly 60 % (33/56) of the publications we analyzed provided no information on their research timeframe or offered results of one year (or one crop cycle) only (Table S3). Among those with specific study periods, thirteen were of short-term studies (≤5 years), and six were of medium-term (between 5 and ≤10 years). Of the four papers that had study durations of more than ten years, only Nunes et al. [49] presented long-term monitoring data of a food product. Others were on farmers’ surveys on their perception of changes over 20 years [59]; the effects of a long-term BGI [48]; and projected benefit-cost-ratio on a 35-year long time horizon [62].

While there are no concrete guidelines on how long an NBS application should be monitored, it has been noted that the values of indicators on success and/or failures of the study objectives could change on a long-term basis [[84], [85], [86]]. For example, Akinnifesi et al. [34] observed that positive effects of multiple agroforestry practices could take up to several years to become apparent but then might be negated shortly afterwards. On the other hand, Kumar et al. [70] reported that the outcomes of a coconut-based agroforestry system varied over time, depending on the development stages of the trees, which then decided the appropriate associated crops. Hence, it can be argued that studies on NBS applications without a proactive temporal monitoring regime risk mischaracterizing their potential and neglecting factors that influence their efficiency over time.

## 5 Economic evaluations of NBS applications

Only a quarter of all analyzed resources in this review (14/56) contained information on economic evaluation of the NBS approaches applied, either in the forms of investment or benefit assessment (Table S3). Six of them presented some information related to the cost required to initiate their respective approach at different levels of detail, ranging from short-term initial support from benefactors [46,61] to breakdowns of individual components of the investments [47,51,52,54]. Reports of income were presented with a variety of units varying from income per capita [63] to a community's aggregated income [34]. In two cases, economic benefits were of hypothetical values like projected benefit-cost-ratio for the next 35 years [62] or in the future scenarios [50]. Only two papers reported both the costs and benefits of the utilized practices with great detail [51,52].

The scarcity of economic evaluation of NBS approaches in our database indicates a general lack of focus on the economic benefits of these practices in peer-reviewed publications. While NBS approaches may not require high levels of funding to be effective [3], it is essential to have continuing financial support for NBS’ implementation until the system can be financially maintained, and monitoring so that their positive impacts are sustained [[87], [88], [89]]. Any farmers and policymakers that would like to invest in efforts to conserve ecosystem services in agricultural settings should understand the vast monetary advantages of intensive and expanding agriculture and hence, create financial incentives that lean on both direct payment and non-financial benefits like livelihood advancement [87].

### 5.1 Incorporation of water benefits

Only in 20 % of all sources reviewed in this study (11/56) were water-related outputs specified and measured (Table S3). Within these publications, increasing water retention by either BGI [40,45,51] or biofertilizers [56,58,90,91] was the most popular approach. Another common method was to reduce the demand for water in food production by altering traditional agricultural practices like applying non-puddling transplanting of rice [54,92] and switching to less water-intensive crops [59].

While a number of factors can affect global food security, water scarcity is perhaps the most impactful and it is expected that a severe food security crisis may occur should there be no substantial changes to future water usage [93]. Such effects are particularly negative in poor countries with lower mitigation/adaptation capacity [93]. In places like the Lower Mekong River Basin (LMRB), where rainfall patterns and intensity are predicted to change and populations are projected to rise significantly in the future, loss of food productivity from rainfed areas must be compensated by increases in extracts from blue water resources [[93], [94], [95], [96]]. For crops that are highly dependent on rainfall like rice, the amount of water needed for their production could be restricted due to the competition for freshwater from industrial and domestic uses [93]. Moreover, water resources in LMRB face not only the effects of climate change but also changes in infrastructure along the river, especially dams and hydropower plants [97]. It is, therefore, essential that NBS applications to enhance food security in this region must incorporate mitigating and/or adapting effects to water scarcity.

### 5.2 Incorporation of biodiversity benefits

Biodiversity was explicitly measured in less than 10 % of the reviewed NBS records (n = 5) (Table S3). Specifically, one stated that intercropping of genetically diverse varieties of rice could result in higher yield per hectare, thus reducing the need to expand planted land to produce the same amount of production [53]. Two other studies focused on species diversity of crops [57] and microbiota diversity in soil [72]. Interestingly, the subject of measured biodiversity in a study of NBS can also be weed diversity, a common target of eradication efforts in agriculture [55]. Their diversity can be used as a soil fertility indicator after applying NBS practices. Moreover, implementation of NBS can ensure functional and species diversity of the study area, and enhance its resilience to climate change [46].

Given the rising popularity of NBS and an increasing number of practices being re-labeled as NBS, there is a need to establish a common set of criteria to identify good practices that comprehensively address environmental challenges and to avoid confusion over what actually can be counted as NBS [98,99]. While different researchers might have different lists of criteria by which they use to classify a specific design as NBS, one of the most common core ideas is to enhance or provide net benefits to biodiversity [99]. Therefore, we urge fellow researchers to follow strictly the requirement of biodiversity gains while evaluate the impacts of their chosen NBS. That means the implementation of NBS in agriculture can only be considered successful if there are simultaneous benefits to food security for the associated populations and to the biodiversity of the targeted ecosystems.

## 6 Recommendations

Considering the growth in demands for food security enhancement policies while maintaining and enhancing ecosystem health, and the rising popularity of NBS, there is a need to identify possible solutions to remove barriers to their implementations. In this paper, we attempt to provide a number of recommendations that correspond to the specific limitations that we highlighted in the previous section.

### 6.1 Shift focus to food utilization, access and stability

While the disproportional focus on volumes of food production is not a new phenomenon, it also suggests that most of the NBS applications reviewed in this study would neglect the need to eliminate the ‘hidden hunger’. This could be the result of the decades-long conflation of increasing food production with ensuring food security [78,79]. As a result, we strongly advocate for a shift from an emphasis on food availability to one on food utilization and access. Specifically, NBS applications to ensure food security in the future need to divert from focusing solely on generic crops production to nutrient and protein-rich food production [82]. Research objectives then should be one of increasing crop nutrient levels [80,81], increasing sustainable animal protein production [[100], [101], [102], [103]], and increasing protein-rich plant production [104].

To achieve this goal, more funding needs to be channeled to future NBS applications that target improvements in macro- and micro-nutrient contents of food products for underprivileged communities as they are the most vulnerable to the effects of climate change [82]. Furthermore, there must be an adequate set of access and utilization indicators to apply alongside production indicators [78,79]. Moreover, there must be an established baseline by which the nutrient needs and major causes of “hidden hunger” in specific regions are identified so that the appropriate NBS can be applied [80,81]. Therefore, it is important to consult local communities, including women and disadvantaged groups, where one plans to implement NBS to safeguard food security. Besides being a sign of respect for their culture and history, incorporating the communities into the design and implementation processes not only ensures equitable distribution of benefits but also helps avoid repeating past mistakes [98].

### 6.2 Develop robust monitoring and evaluation systems

As the effects of applied NBS are not spatially and temporally uniform [85,86], it is essential that studies on their potential to improve food security must have monitoring frameworks that can take into account the changing dynamics of the approaches in question [25,84]. As NBS are expected to have multiple co-benefits, e.g., farmers, consumers, and the environment in a food production system, the required frameworks must be based on an adequate list of indicators that can measure the efficiency of NBS and are applicable to a number of different such as production volumes, product quality, financial benefits, ecosystem health, and biodiversity [25,85,86]. Moreover, the success of a monitoring regime also depends on the quality and quantity of available data as well as how they are managed. In this case, it has been suggested that ecological data such as biodiversity, and soil and water qualities should be treated and funded equally throughout the lifetime of a project [105].

Besides a comprehensive framework, it is also essential that the monitoring regimes are extensive enough to capture the true effects of NBS. An adequate monitoring period allows the distinction of short-term and long-term effects [86,106] and helps produce robust analyses that can strengthen the evidence-based knowledge of NBS and food security [107]. While examples of medium- or long-term monitoring on NBS and food security were rare, the fact that some lasted up to 30 years, e.g. Refs. [34,41,48,49], is encouraging and their designs could be used as templates for future research.

Industrialized agricultural models of intensive food production with heavy dependence on chemical fertilizers and pesticides, and on water from irrigation have resulted in significant damage to ecosystems around the world [17,18] but still remain financially advantageous over CA. Therefore, it is important to establish a design framework and evaluation of NBS approaches in which economic costs and benefits are explicitly assessed and reported. Such information is necessary to comprehensively inform policymakers and farmers who will decide upon implementing the recommended approaches after scientific knowledge has been published [89,107]. Moreover, financial benefits are important to ensure public support and long-term engagement, especially for underprivileged communities and women [34,[107], [108], [109]].

### 6.3 Implement financial support systems

Despite the potential of preventing major losses of ecosystem services while maintaining human benefits at the same time, levels of financial support for NBS have been reported to be inadequate and would fail to meet the required level to achieve international changes in biodiversity and other targets [110]. Better funding and human resources were found to be the key components to successfully integrate knowledge generation, policy-making, and ecosystem service conservation in NBS intervention [88,89]. Although researching and implementing NBS typically depend on public funding, governmental financial support in this field is still limited [111,112]. Therefore, it has been recommended that private finance should be mobilized and blended with government support to address this financial shortfall, and to create public incentives to contribute to sustainable development. This type of innovative finance is expected to make NBS projects more effective and efficient while concurrently providing the private sector financial benefits [110,113,114]. Nonetheless, we would like to reassert that implications of government policies and strategies on the implementation of NBS were not reviewed in this study. There have been examples in which national and local governments took an active role in investing and supporting NBS applications, such as the Bangladeshi government's “Hilsha Fishery Management Action Plan” [115] and the Rio de Janeiro's “RJ Integrated Agroecosystem Management” [89]. Therefore, it is also recommended that subsequent reviews can target government actions, policies and directives, and their implications on the implementations of NBS in the agriculture sector to provide a more holistic understanding of this research field.

### 6.4 Incorporate water security

Agriculture is the biggest user of water in virtually all countries in the world [96,116] and the effects of climate change, and the growing population on water resources available to agricultural activities can severely impact food production [93,117]. As a result, efforts to mitigate such effects are required at both global and regional scales [118] and improving water productivity in agriculture is the crucial response to resolve the joined issue of water and food security [93,119]. Meanwhile, numerous examples of NBS practices have been found to be effective in improving water security around the world (see Cassin et al. [120] and references therein) and many of the reviewed papers in this article indicated significant and positive outcomes in both food production and water productivity, e.g. Refs. [40,45], and [58].

Thus, it is essential that future applications of NBS incorporate the requirement to monitor water productivity throughout the course of the project. While there has been much debate on the specific methods to measure water productivity in agriculture [121,122], common measurements like soil moisture were reported to be an efficient indicator of overall water use efficiency and therefore, could be included as a required measurement in designing NBS approaches [123,124].

### 6.5 Enforce biodiversity component

It is also crucial that for a practice to be described as a NBS, it needs to have specific and measured biodiversity outputs [98,99]. Moreover, said biodiversity measurements must be carefully designed to maintain or restore ecosystem services that may have been adversely affected by agricultural practices. For example, applications of agroforestry have been frequently reported as beneficial to biodiversity but, if done incorrectly, such as plantations of non-native trees and afforestation of natural grassland, can actually diminish the natural biodiversity of the study area [98]. Having a well-defined and established list of biodiversity indicators in place can help identify and eliminate attempts to ‘greenwash’ an ineffective practice, like monoculture tree plantations, easily from consideration [99]. Furthermore, it should be made clear in studies on NBS that producing desired food security benefits and biodiversity need to be accompanied by the protection of neighboring ecosystems so that the negative impacts cannot be simply shifted somewhere else to satisfy food production demand [98,125,126].

Ideally, one should apply the key criteria described by Seddon et al. [98] and Sowińska-Świerkosz et al. [99] before designing and implementing a set of NBS approaches to enhance the food security of a specific population or location. Specifically, one needs to be able to 1) identify the baseline biodiversity of the research area, 2) select a diverse mix of crops and animals as research subjects, and 3) set quantitative biodiversity goals that can be monitored and managed over time.

## 7 Conclusion

The findings in this literature review indicate that NBS practices have the potential to improve multiple dimensions of food security individually and simultaneously, but past studies on this subject contained multiple significant limitations that can severely impact their applications and effectiveness. Henceforth, to better support the implementations of NBS on food security research and planning, we provided a number of recommendations that strategically target the identified barriers.

The results of the analyzed publications particularly indicate that a wide variety of NBS approaches can be employed to enhance food security while providing additional benefits such as increases in water security, soil health, and nutrient content. Moreover, these practices can be applied either singly or in combinations with others to achieve the desired outcomes. However, such benefits were not uniformly available across all food security dimensions as there was a strong bias towards using NBS to improve the volumes of food production and against their application to enhance food access and utilization. Most of the reviewed sources were on studies with short-term periods and no economic evaluations of the NBS approaches involved. The key components of simultaneous environmental benefits, especially biodiversity measures, were largely missing in most studies.

We, therefore, suggest that future research on NBS to safeguard food security needs to shift focus to food access and utilization by improving nutrient contents in crops, and sustainable plant and animal protein production. These studies should be based on careful consultation with local communities, addressing their nutrient needs by using indicators that can be easily measured and managed. Successful implementation of NBS has to come with a systematic monitoring regime and a robust and diverse financial support system. Finally, biodiversity and other environmental benefits must be incorporated into the planning and design of an NBS study so that they can meet the criterion of “simultaneously providing human well-being and biodiversity benefits”.

## Data availability statement

The data that support the findings of this study have not been deposited into a publicly available repository and are available from the corresponding author, [HHL], upon reasonable request.

## Ethics statement

Review and/or approval of an ethics committee was not applicable to this study because all findings and analyses were based exclusively on published materials.

## CRediT authorship contribution statement

**Hoang Minh Nguyen:** Writing – original draft, Visualization, Methodology, Investigation, Formal analysis, Data curation, Conceptualization. **Huu Loc Ho:** Writing – review & editing, Supervision, Resources, Project administration, Methodology, Investigation, Funding acquisition, Conceptualization. **M.S. Babel:** Writing – review & editing, Conceptualization. **Natthachet Tangdamrongsub:** Writing – review & editing, Validation. **Sushil Kumar Himanshu:** Writing – review & editing. **Perrine Hamel:** Writing – review & editing, Validation. **Edward Park:** Writing – review & editing, Validation.

## Declaration of competing interest

The authors declare that they have no known competing financial interests or personal relationships that could have appeared to influence the work reported in this article.
