# Supplementary material for: Nature-based solutions for improving food security: A systematic global review
Source: Heliyon. 2024 Aug 14;10(16):e36082. doi: 10.1016/j.heliyon.2024.e36082 (PMC11378918; doi:10.1016/j.heliyon.2024.e36082)
Supplement: Multimedia component 1 [file mmc1.docx]

**Table S1.** Targeted food security dimensions and research subjects of the publications reviewed in this study

| **Author(s) and Year of Publication** | **Continent** | **Targeted Food Security Dimension** | | | | **Food Products** | | |
| --- | --- | --- | --- | --- | --- | --- | --- | --- |
|  |  | **Availability** | **Access** | **Utilization** | **Stability** | **Crops** | **Livestock** | **Aquaculture** |
| **Global North Studies** | | | | | | | | |
| Barbeau et al. [1] | North America | x |  |  |  | x |  |  |
| Cope et al. [2] | Europe | x |  |  |  | x |  |  |
| Cowden et al. [3] | Europe | x |  | x |  | x |  |  |
| Głowacka et al. [4] | Europe | x |  | x | x | x |  |  |
| Goldstein et al. [5] | North America | x |  |  | x | x |  |  |
| Li et al. [6] | Europe | x |  |  |  |  |  | x |
| N'Dayegamiye et al. [7] | North America | x |  | x |  | x |  |  |
| Smith et al. [8] | North America | x |  |  | x | x |  |  |
| Smith et al. [9] | North America |  |  | x |  | x |  |  |
| Weih et al. [10] | Europe | x |  |  | x | x |  |  |
| **Global South Studies** | | | | | | | | |
| Abdul Rahman et al. [11] | Africa | x |  |  | x | x |  |  |
| Ahammad et al. [12] | Asia | x | x | x | x | x | x | x |
| Ahmed et al. [13] | Asia | x | x | x |  | x |  | x |
| Akinnifesi et al. [14] | Africa | x | x |  | x | x |  |  |
| Are et al. [15] | Africa | x |  |  | x | x |  |  |
| Balamatti et al. [16] | Asia | x |  |  |  | x |  |  |
| Bell et al. [17] | Asia | x |  |  |  | x |  |  |
| Bhattacharyya et al. [18] | Asia | x |  |  |  | x |  |  |
| Bitew et al. [19] | Africa | x |  |  | x | x |  |  |
| Brilhante et al. [20] | Africa |  | x | x |  | x |  |  |
| Brouder et al. [21] | Africa | x |  |  |  | x |  |  |
| Brouwer et al. [22] | Africa | x |  |  |  | x |  |  |

**Table S1.** (cont.)

| **Author(s) and Year of Publication** | **Continent** | **Targeted Food Security Dimension** | | | | **Food Products** | | |
| --- | --- | --- | --- | --- | --- | --- | --- | --- |
|  |  | **Availability** | **Access** | **Utilization** | **Stability** | **Crops** | **Livestock** | **Aquaculture** |
| Chan et al. [23] | Asia | x |  |  |  | x |  |  |
| Costa et al. [24] | Latin America | x | x |  |  | x | x |  |
| Dineshkumar et al. [25] | Asia | x |  | x |  | x |  |  |
| d'Oultremont et al. [26] | Asia | x |  |  |  | x |  | x |
| Huang et al. [27] | Africa | x |  |  | x | x |  |  |
| Islam et al. [28] | Asia | x |  |  |  | x |  |  |
| Kumar et al. [29] | Asia | x |  |  | x | x |  |  |
| Lei et al. [30] | Asia |  | x |  | x | x | x |  |
| Lenka et al. [31] | Asia | x |  |  | x | x |  |  |
| Mishra et al. [32] | Asia | x |  |  |  | x |  | x |
| Nunes et al. [33] | Latin America | x |  |  |  | x |  |  |
| Pandit et al. [34] | Asia |  | x |  | x | x | x |  |
| Pereira et al. [35] | Latin America | x |  |  |  | x |  |  |
| Peter et al. [36] | Africa | x |  |  |  | x |  |  |
| Rahman et al. [37] | Asia |  | x |  | x |  |  | x |
| Rashid et al. [38] | Asia | x |  |  |  | x |  |  |
| Risna et al. [39] | Asia | x |  |  | x | x |  |  |
| Rosa et al. [40] | Latin America | x |  |  |  | x |  |  |
| Satori et al. [41] | Africa | x |  |  |  | x |  |  |
| Shamsudduha et al. [42] | Asia | x |  |  | x | x |  |  |
| Sida et al. [43] | Africa | x |  |  |  | x |  |  |
| Singh et al. [44] | Asia | x |  |  |  | x |  |  |
| Swamy et al. [45] | Asia | x |  |  |  | x |  |  |
| Thorlakson et al. [46] | Africa | x |  |  | x | x | x |  |
| Vaidya et al. [47] | Asia | x |  |  |  | x |  |  |
| Wadne et al. [48] | Asia | x |  |  |  | x |  |  |
| Wang et al. [49] | Asia | x |  |  | x |  | x |  |

**Table S1.** (cont.)

| **Author(s) and Year of Publication** | **Continent** | **Targeted Food Security Dimension** | | | | **Food Products** | | |
| --- | --- | --- | --- | --- | --- | --- | --- | --- |
|  |  | **Availability** | **Access** | **Utilization** | **Stability** | **Crops** | **Livestock** | **Aquaculture** |
| Zhang et al. [50] | Asia | x |  |  | x | x |  |  |
| Zhu et al. [51] | Asia | x |  |  |  | x |  |  |
| **Transcontinental Studies** | | | | | | | | |
| Garrett et al. [52] | Global | x |  |  | x | x |  |  |
| Li et al. [53] | Global | x |  |  |  | x |  |  |
| Mezeli et al. [54] | Global | x |  |  |  | x |  |  |
| Tonitto et al. [55] | Global | x |  |  | x | x |  |  |
| van der Schatte Olivier et al. [56] | Global | x |  |  |  |  |  | x |

**Table S2.** Categories of the Nature-based solutions applied in the publications reviewed in this study

| **Author(s) and Year of Publication** | **NBS Categories** | | | | | | |
| --- | --- | --- | --- | --- | --- | --- | --- |
|  | **Agroforestry** | **Conservation Agriculture** | **Green/blue Infrastructure** | **Natural Supplementation** | **Sustainable Husbandry** | **Sustainable Aquaculture** | **Others** |
| **Global North Studies** | | | | | | | |
| Barbeau et al. [1] | x |  | x |  |  |  |  |
| Cope et al. [2] |  |  |  |  |  |  | x |
| Cowden et al. [3] |  | x |  |  |  |  |  |
| Głowacka et al. [4] |  | x |  |  |  |  |  |
| Goldstein et al. [5] |  | x |  | x |  |  | x |
| Li et al. [6] |  |  |  |  |  | x |  |
| N'Dayegamiye et al. [7] |  | x |  |  |  |  |  |
| Smith et al. [8] |  | x |  |  |  |  |  |
| Smith et al. [9] |  | x |  |  |  |  |  |
| Weih et al. [10] |  | x |  |  |  |  |  |
| **Global South Studies** | | | | | | | |
| Abdul Rahman et al. [11] |  |  |  | x |  |  |  |
| Ahammad et al. [12] | x | x | x |  | x | x |  |
| Ahmed et al. [13] |  |  |  |  |  | x | x |
| Akinnifesi et al. [14] | x |  |  |  |  |  |  |
| Are et al. [15] |  | x | x |  |  |  |  |
| Balamatti et al. [16] |  |  |  | x |  |  |  |
| Bell et al. [17] |  | x |  |  |  |  |  |
| Bhattacharyya et al. [18] |  | x | x |  |  |  |  |
| Bitew et al. [19] |  | x |  |  |  |  |  |
| Brilhante et al. [20] |  | x |  |  |  |  |  |
| Brouder et al. [21] |  | x |  |  |  |  |  |
| Brouwer et al. [22] |  |  |  | x |  |  |  |

**Table S2.** (cont.)

| **Author(s) and Year of Publication** | **NBS Categories** | | | | | | |
| --- | --- | --- | --- | --- | --- | --- | --- |
|  | **Agroforestry** | **Conservation Agriculture** | **Green/blue Infrastructure** | **Natural Supplementation** | **Sustainable Husbandry** | **Sustainable Aquaculture** | **Others** |
| Chan et al. [23] |  | x |  |  |  |  |  |
| Costa et al. [24] | x |  |  |  | x |  |  |
| Dineshkumar et al. [25] |  |  |  | x |  |  |  |
| d'Oultremont et al. [26] |  |  |  |  |  | x | x |
| Huang et al. [27] |  |  | x |  |  |  |  |
| Islam et al. [28] |  | x |  |  |  |  |  |
| Kumar et al. [29] | x |  |  |  |  |  |  |
| Lei et al. [30] | x |  | x |  | x |  |  |
| Lenka et al. [31] |  |  | x |  |  |  |  |
| Mishra et al. [32] |  |  | x |  |  | x |  |
| Nunes et al. [33] |  | x |  |  |  |  |  |
| Pandit et al. [34] | x |  |  |  | x |  |  |
| Pereira et al. [35] |  |  |  | x |  |  |  |
| Peter et al. [36] |  |  |  |  |  |  | x |
| Rahman et al. [37] | x |  |  |  |  | x |  |
| Rashid et al. [38] |  | x |  |  |  |  |  |
| Risna et al. [39] |  |  | x |  |  |  |  |
| Rosa et al. [40] |  |  |  | x |  |  |  |
| Satori et al. [41] |  |  |  |  |  |  | x |
| Shamsudduha et al. [42] |  |  | x |  |  |  |  |
| Sida et al. [43] | x |  |  |  |  |  |  |
| Singh et al. [44] |  | x | x |  |  |  |  |
| Swamy et al. [45] |  |  |  | x |  |  |  |
| Thorlakson et al. [46] | x |  |  |  | x |  |  |
| Vaidya et al. [47] |  |  |  | x |  |  |  |

**Table S2.** (cont.)

| **Author(s) and Year of Publication** | **NBS Categories** | | | | | | |
| --- | --- | --- | --- | --- | --- | --- | --- |
|  | **Agroforestry** | **Conservation Agriculture** | **Green/blue Infrastructure** | **Natural Supplementation** | **Sustainable Husbandry** | **Sustainable Aquaculture** | **Others** |
| Wadne et al. [48] |  |  |  | x |  |  |  |
| Wang et al. [49] |  |  |  |  | x |  |  |
| Zhang et al. [50] |  |  |  | x |  |  |  |
| Zhu et al. [51] |  | x |  |  |  |  |  |
| **Transcontinental Studies** | | | | | | | |
| Garrett et al. [52] |  |  |  |  |  |  | x |
| Li et al. [53] |  | x |  |  |  |  |  |
| Mezeli et al. [54] |  |  |  | x |  |  |  |
| Tonitto et al. [55] |  | x |  |  |  |  |  |
| van der Schatte Olivier et al. [56] |  |  | x |  |  | x |  |

**Table S3.** Additional characteristics of publications reviewed in this paper

| **Author(s) and Year of Publication** | **Study Duration**^a^ | **Economic Assessment**^b^ | **Other Benefits**^c^ |
| --- | --- | --- | --- |
| **Global North Studies** | | | |
| Barbeau et al. [1] |  |  |  |
| Cope et al. [2] |  |  |  |
| Cowden et al. [3] |  |  |  |
| Głowacka et al. [4] | 3 years |  |  |
| Goldstein et al. [5] | 3 to 6 years |  | Increase in carbon sequestration |
|  |  |  | Increase in nutrient storage |
|  |  |  | Increase in microbial biomass |
| Li et al. [6] |  |  |  |
| N'Dayegamiye et al. [7] |  |  |  |
| Smith et al. [8] | 3 years |  | Increase in biodiversity |
|  |  |  | Increase in nutrient storage |
| Smith et al. [9] |  |  |  |
| Weih et al. [10] | 2 years |  |  |
| **Global South Studies** | | | |
| Abdul Rahman et al. [11] | 2 years |  | Increase in carbon sequestration |
|  |  |  | Increase in nutrient storage |
|  |  |  | Increase in biodiversity |
| Ahammad et al. [12] |  | Initial financial support | Protection against extreme weather events |
|  |  |  | Increase in biodiversity |
| Ahmed et al. [13] |  | Benefit analysis |  |
| Akinnifesi et al. [14] | Up to 10 years | Benefit analysis |  |
| Are et al. [15] | 3 years |  | Reduction in soil loss |
|  |  |  | Increase in carbon sequestration |
|  |  |  | Increase in water storage |

^a^: Blank cells mean the study period was one year or within one crop cycle

^b^: Blank cells mean no economic assessments were conducted in this study

^c^: Blank cells mean no benefits besides food security were identified

**Table S3.** (cont.)

| **Author(s) and Year of Publication** | **Study Duration**^a^ | **Economic Assessment**^b^ | **Other Benefits**^c^ |
| --- | --- | --- | --- |
| Balamatti et al. [16] |  |  | Increase in water storage |
| Bell et al. [17] | 10 years | Cost analysis | Increase in carbon sequestration |
|  |  |  | Increase in nutrient storage |
|  |  |  | Reduction in water demand |
|  |  |  | Increase in microbial biomass |
| Bhattacharyya et al. [18] |  | Benefit analysis |  |
| Bitew et al. [19] | 2 years |  | Reduction in land demand |
| Brilhante et al. [20] |  |  | Resistance against climate change |
| Brouder et al. [21] | Up to 8 years |  |  |
| Brouwer et al. [22] |  |  | Increase in water storage |
|  |  |  | Increase in nutrient storage |
| Chan et al. [23] |  |  |  |
| Costa et al. [24] | 7 years | Cost analysis | Increase in carbon sequestration |
|  |  |  | Reduction in resources demand |
|  |  |  | Reduction in land demand |
| Dineshkumar et al. [25] |  |  | Increase in microbial biomass |
|  |  |  | Increase in nutrient storage |
| d'Oultremont et al. [26] |  |  |  |
| Huang et al. [27] | 10 years |  | Increase in nutrient storage |
| Islam et al. [28] |  |  | Reduce water demand |
| Kumar et al. [29] |  |  | Increase in carbon sequestration |
|  |  |  | Accelerate nutrient cycling processes |
| Lei et al. [30] | 20 years | Benefit analysis | Reduction in water demand |
| Lenka et al. [31] | 2 years |  | Reduction in soil loss |
|  |  |  | Increase in nutrient storage |

^a^: Blank cells mean the study period was one year or within one crop cycle

^b^: Blank cells mean no economic assessments were conducted in this study

^c^: Blank cells mean no benefits besides food security were identified

**Table S3.** (cont.)

| **Author(s) and Year of Publication** | **Study Duration**^a^ | **Economic Assessment**^b^ | **Other Benefits**^c^ |
| --- | --- | --- | --- |
| Mishra et al. [32] | 3 years | Cost and benefit analyses | Increase in water storage |
| Nunes et al. [33] | 17 years |  |  |
| Pandit et al. [34] |  | Benefit analysis |  |
| Pereira et al. [35] |  |  |  |
| Peter et al. [36] |  |  |  |
| Rahman et al. [37] | (Projected) 35 years | Benefit-cost-ratio analysis | Protection against extreme weather events |
| Rashid et al. [38] | 3 years | Cost and benefit analyses |  |
|  |  | Benefit-cost-ratio analysis |  |
| Risna et al. [39] |  |  |  |
| Rosa et al. [40] |  |  |  |
| Satori et al. [41] |  |  | Resistance against climate change |
| Shamsudduha et al. [42] | Up to 30 years |  | Increase in water supply |
| Sida et al. [43] | 3 years |  | Protection against extreme weather events |
| Singh et al. [44] | 2 years |  | Increase in water storage |
|  |  |  | Reduction in soil loss |
| Swamy et al. [45] |  |  | Increase in nutrient uptake |
| Thorlakson et al. [46] |  | Initial financial support | Reduction in soil loss |
|  |  |  | Reduction in extreme coping methods |
| Vaidya et al. [47] | 3 years |  | Increase in water storage |
|  |  |  | Increase in nutrient storage |
| Wadne et al. [48] |  |  | Increase in water storage |
|  |  |  | Increase in nutrient storage |
| Wang et al. [49] |  | Benefit analysis | Maintaining and improving grassland ecosystem |
| Zhang et al. [50] | 2 years |  | Increase in biodiversity |
| Zhu et al. [51] |  | Benefit analysis | Increase in biodiversity |

^a^: Blank cells mean the study period was one year or within one crop cycle

^b^: Blank cells mean no economic assessments were conducted in this study

^c^: Blank cells mean no benefits besides food security were identified

**Table S3.** (cont.)

| **Author(s) and Year of Publication** | **Study Duration**^a^ | **Economic Assessment**^b^ | **Other Benefits**^c^ |
| --- | --- | --- | --- |
| **Transcontinental Studies** | | | |
| Garrett et al. [52] |  |  | Increase in crop resistance to pests |
| Li et al. [53] |  |  |  |
| Mezeli et al. [54] |  |  |  |
| Tonitto et al. [55] |  |  | Increase in nutrient storage |
| van der Schatte Olivier et al. [56] |  |  | Increase in carbon sequestration |

^a^: Blank cells mean the study period was one year or within one crop cycle

^b^: Blank cells mean no economic assessments were conducted in this study

^c^: Blank cells mean no benefits besides food security were identified

**REFERENCES**

[1] C.D. Barbeau, M.J. Wilton, M. Oelbermann, J.D. Karagatzides, L.J.S. Tsuji, Local food production in a subarctic Indigenous community: The use of willow (*Salix* spp.) windbreaks to increase the yield of intercropped potatoes (*Solanum tuberosum*) and bush beans (*Phaseolus vulgaris*), Int. J. Agric. Sustain., 16 (1) (2018) 29 - 39, https://doi.org/10.1080/14735903.2017.1400713

[2] J.E. Cope, G.J. Norton, T.S. George, A.C. Newton, Identifying potential novel resistance to the foliar disease ‘Scald’ (*Rhynchosporium commune*) in a population of Scottish Bere barley landrace (*Hordeum vulgare* L.), J. Plant Dis. Prot., 128 (4) (2021) 999 - 1012, https://doi.org/10.1007/s41348-021-00470-x

[3] R.J. Cowden, A.N. Shah, L.M. Lehmann, L.P. Kiær, C.B. Henriksen, B.B. Ghaley, Nitrogen fertilizer effects on pea–barley intercrop productivity compared to sole crops in Denmark, Sustainability, 12 (22) (2020) 9335, https://doi.org/10.3390/su12229335

[4] A. Głowacka, B. Szostak, R. Klebaniuk, A. Kiełtyka-Dadasiewicz, Is Strip Cropping an Effective Way for Maize Biofortyfication?, J. Chem., 2018 (2018) 1 - 9, https://doi.org/10.1155/2018/1601920

[5] W.A. Goldstein, H.H. Koepf, C.J. Koopmans, Biodynamic preparations, greater root growth and health, stress resistance, and soil organic matter increases are linked, Open Agric., 4 (1) (2019) 187 - 202, https://doi.org/10.1515/opag-2019-0018

[6] M. Li, M.D. Callier, J.-P. Blancheton, A. Galès, S. Nahon, S. Triplet, T. Geoffroy, C. Menniti, E. Fouilland, E. Roque D'Orbcastel, Bioremediation of fishpond effluent and production of microalgae for an oyster farm in an innovative recirculating integrated multi-trophic aquaculture system, Aquaculture, 504 (2019) 314 - 325, https://doi.org/10.1016/j.aquaculture.2019.02.013

[7] A. N'Dayegamiye, J.K. Whalen, G. Tremblay, J. Nyiraneza, M. Grenier, A. Drapeau, M. Bipfubusa, The benefits of legume crops on corn and wheat yield, nitrogen nutrition, and soil properties improvement, Agron. J., 107 (5) (2015) 1653 - 1665, https://doi.org/10.2134/agronj14.0416

[8] R.G. Smith, K.L. Gross, G.P. Robertson, Effects of crop diversity on agroecosystem function: crop yield response, Ecosystems, 11 (3) (2008) 355 - 366, https://doi.org/10.1007/s10021-008-9124-5

[9] E.G. Smith, H.H. Janzen, B.H. Ellert, Effect of fertilizer and cropping system on grain nutrient concentrations in spring wheat, Can. J. Plant. Sci., 98 (1) (2017) 125 - 131, https://doi.org/10.1139/cjps-2017-0079

[10] M. Weih, A.J. Karley, A.C. Newton, L.P. Kiær, C. Scherber, D. Rubiales, E. Adam, J. Ajal, J. Brandmeier, S. Pappagallo, Grain yield stability of cereal-legume intercrops is greater than sole crops in more productive conditions, Agriculture, 11 (3) (2021) 255, https://doi.org/10.3390/agriculture11030255

[11] N. Abdul Rahman, A. Larbi, A. Opoku, F.M. Tetteh, I. Hoeschle-Zeledon, Corralling, planting density, and N fertilizer rate effect on soil properties, weed diversity, and maize yield, Agroecol. Sustain. Food Syst., 43 (3) (2019) 243 - 260, https://doi.org/10.1080/21683565.2018.1516264

[12] R. Ahammad, P. Nandy, P. Husnain, Unlocking ecosystem based adaptation opportunities in coastal Bangladesh, J. Coast. Conservation, 17 (4) (2013) 833 - 840, https://doi.org/10.1007/s11852-013-0284-x

[13] N. Ahmed, S.W. Bunting, S. Rahman, C.J. Garforth, Community‐based climate change adaptation strategies for integrated prawn–fish–rice farming in Bangladesh to promote social–ecological resilience, Rev. Aquac., 6 (1) (2014) 20-35, https://doi.org/10.1111/raq.12022

[14] F.K. Akinnifesi, P.W. Chirwa, O.C. Ajayi, G. Sileshi, P. Matakala, F.R. Kwesiga, H. Harawa, W. Makumba, Contributions of agroforestry research to livelihood of smallholder farmers in Southern Africa: 1. Taking stock of the adaptation, adoption and impact of fertilizer tree options, Agric. J., 3 (1) (2008) 58 - 75, https://medwelljournals.com/abstract/?doi=aj.2008.58.75

[15] K.S. Are, S.O. Oshunsanya, G.A. Oluwatosin, Changes in soil physical health indicators of an eroded land as influenced by integrated use of narrow grass strips and mulch, Soil Tillage Res., 295 (2018) 269 - 280, https://doi.org/10.1016/j.still.2018.08.009

[16] A. Balamatti, C.S. Chandra, Impact of tank silt on soil fertility–A study in Mysuru District, Indian J. Dryland Agric. Res. Dev., 33 (2) (2018) 14 - 21, http://dx.doi.org/10.5958/2231-6701.2018.00015.5

[17] R.W. Bell, M.E. Haque, M. Jahiruddin, M.M. Rahman, M. Begum, M.M. Miah, M.A. Islam, M.A. Hossen, N. Salahin, T. Zahan, Conservation agriculture for rice-based intensive cropping by smallholders in the eastern Gangetic plain, Agriculture, 9 (1) (2018) 5, https://doi.org/10.3390/agriculture9010005

[18] R. Bhattacharyya, Z. Yi, L. Yongmei, T. Li, M. Panomtaranichagul, S. Peukrai, D.C. Thu, T.H. Cuong, T.T. Toan, B. Jankauskas, Effects of biological geotextiles on aboveground biomass production in selected agro-ecosystems, Field Crops Res., 126 (2012) 23 - 36, https://doi.org/10.1016/j.fcr.2011.09.006

[19] Y. Bitew, G. Alemayehu, E. Adgo, A. Assefa, Competition, production efficiency and yield stability of finger millet and legume additive design intercropping, Renew. Agric. Food Syst., 36 (1) (2021) 108 - 119, https://doi.org/10.1017/S1742170520000101

[20] M. Brilhante, E. Varela, A. P. Essoh, A. Fortes, M.C. Duarte, F. Monteiro, V. Ferreira, A.M. Correia, M.P. Duarte, M.M. Romeiras, Tackling food insecurity in Cabo Verde Islands: The nutritional, agricultural and environmental values of the legume species, Foods, 10 (2) (2021) 206, https://doi.org/10.3390/foods10020206

[21] S.M. Brouder, H. Gomez-Macpherson, The impact of conservation agriculture on smallholder agricultural yields: A scoping review of the evidence, Agric., Ecosyst. Environ., 187 (2014) 11 - 32, https://doi.org/10.1016/j.agee.2013.08.010

[22] J. Brouwer, J.M. Powell, Increasing nutrient use efficiency in West-African agriculture: the impact of micro-topography on nutrient leaching from cattle and sheep manure, Agric., Ecosyst. Environ., 71 (1 - 3) (1998) 229 - 239, https://doi.org/10.1016/S0167-8809(98)00143-1

[23] C. Chan, B. Sipes, A. Ayman, X. Zhang, P. LaPorte, F. Fernandes, A. Pradhan, J. Chan-Dentoni, P. Roul, Efficiency of conservation agriculture production systems for smallholders in rain-fed uplands of India: A transformative approach to food security, Land, 6 (3) (2017) 58, https://doi.org/10.3390/land6030058

[24] M.P. Costa, J.C. Schoeneboom, S.A. Oliveira, R.S. Vinas, G.A. de Medeiros, A socio-eco-efficiency analysis of integrated and non-integrated crop-livestock-forestry systems in the Brazilian Cerrado based on LCA, J. Clean. Prod., 171 (2018) 1460 - 1471, https://doi.org/10.1016/j.jclepro.2017.10.063

[25] R. Dineshkumar, J. Subramanian, J. Gopalsamy, P. Jayasingam, A. Arumugam, S. Kannadasan, P. Sampathkumar, The impact of using microalgae as biofertilizer in maize (*Zea mays* L.), Waste Biomass Valorization, 10 (2019) 1101 - 1110, https://doi.org/10.1007/s12649-017-0123-7

[26] T. d'Oultremont, A.P. Gutierrez, A multitrophic model of a rice–fish agroecosystem: II. Linking the flooded rice–fishpond systems, Ecol. Model., 155 (2 - 3) (2002) 159 - 176, https://doi.org/10.1016/S0304-3800(02)00130-8

[27] Z. Huang, S.O. Oshunsanya, Y. Li, H. Yu, K.S. Are, Vetiver grass hedgerows significantly trap P but little N from sloping land: Evidenced from a 10-year field observation, Agric. Ecosyst. Environ., 281 (2019) 72 - 80, https://doi.org/10.1016/j.agee.2019.05.005

[28] S. Islam, M.K. Gathala, T.P. Tiwari, J. Timsina, A.M. Laing, S. Maharjan, A.K. Chowdhury, P.M. Bhattacharya, T. Dhar, B. Mitra, Conservation agriculture based sustainable intensification: increasing yields and water productivity for smallholders of the Eastern Gangetic Plains, Field Crops Res., 238 (2019) 1 - 17, https://doi.org/10.1016/j.fcr.2019.04.005

[29] B.M. Kumar, T.K. Kunhamu, Nature-Based Solutions in Agriculture: A Review of the Coconut (*Cocos nucifera* L.)-Based Farming Systems in Kerala,“the Land of Coconut Trees”, Nature-Based Solutions, 2 (2022) 100012, https://doi.org/10.1016/j.nbsj.2022.100012

[30] Y. Lei, H. Zhang, F. Chen, L. Zhang, How rural land use management facilitates drought risk adaptation in a changing climate—A case study in arid northern China, Sci. Total Environ., 550 (2016) 192 - 199, https://doi.org/10.1016/j.scitotenv.2016.01.098

[31] N.K. Lenka, K.K. Satapathy, R. Lal, R.K. Singh, N.A.K. Singh, P.K. Agrawal, P. Choudhury, A. Rathore, Weed strip management for minimizing soil erosion and enhancing productivity in the sloping lands of north-eastern India, Soil Tillage Res., 170 (2017) 104 - 113, https://doi.org/10.1016/j.still.2017.03.012

[32] A. Mishra, R.K. Mohanty, Productivity enhancement through rice–fish farming using a two-stage rainwater conservation technique, Agric. Water Manage., 67 (2004) 119 - 131, https://doi.org/10.1016/j.agwat.2004.02.003

[33] R.d.S. Nunes, D.M.G. de Sousa, W.J. Goedert, L.E.Z. de Oliveira, P.S. Pavinato, T.D. Pinheiro, Distribution of soil phosphorus fractions as a function of long-term soil tillage and phosphate fertilization management, Front. Earth Sci., 8 (2020) 350, https://doi.org/10.3389/feart.2020.00350

[34] B.H. Pandit, I. Nuberg, K.K. Shrestha, E. Cedamon, S.M. Amatya, B. Dhakal, R.P. Neupane, Impacts of market-oriented agroforestry on farm income and food security: insights from Kavre and Lamjung districts of Nepal, Agrofor. Syst., 93 (4) (2019) 1593 - 1604, https://doi.org/10.1007/s10457-018-0273-z

[35] N.C.M. Pereira, F.S. Galindo, R.P.D. Gazola, E. Dupas, P.A.L. Rosa, E.S. Mortinho, M.C.M. Teixeira Filho, Corn yield and phosphorus use efficiency response to phosphorus rates associated with plant growth promoting bacteria, Front. Environ. Sci., 8 (2020) 40, https://doi.org/10.3389/fenvs.2020.00040

[36] B.G. Peter, L.M. Mungai, J.P. Messina, S.S. Snapp, Nature-based agricultural solutions: scaling perennial grains across Africa, Environ. Res., 159 (2017) 283 - 290, https://doi.org/10.1016/j.envres.2017.08.011

[37] M.M. Rahman, M.A. Mahmud, Economic feasibility of mangrove restoration in the Southeastern Coast of Bangladesh, Ocean Coastal Manage., 161 (2018) 211 - 221, https://doi.org/10.1016/j.ocecoaman.2018.05.009

[38] M.H. Rashid, J. Timsina, N. Islam, S. Islam, Tillage and residue-management effects on productivity, profitability and soil properties in a rice-maize-mungbean system in the Eastern Gangetic Plains, J. Crop. Improv., 33 (5) (2019) 683 - 710, https://doi.org/10.1080/15427528.2019.1661056

[39] R.A. Risna, H.A. Rustini, D. Buchori, D.O. Pribadi, Subak, a Nature-based Solutions Evidence from Indonesia, IOP Conf. Ser.: Earth Environ. Sci., 959 (1) (2022) 012030, https://doi.org/10.1088/1755-1315/959/1/012030

[40] P.A.L. Rosa, E.S. Mortinho, A. Jalal, F.S. Galindo, S. Buzetti, G.C. Fernandes, M. Barco Neto, P.S. Pavinato, M.C.M. Teixeira Filho, Inoculation with growth-promoting bacteria associated with the reduction of phosphate fertilization in sugarcane, Front. Environ. Sci., 8 (2020) 32, https://doi.org/10.3389/fenvs.2020.00032

[41] D. Satori, C. Tovar, A. Faruk, E. Hammond Hunt, G. Muller, C. Cockel, N. Kühn, I.J. Leitch, E. Lulekal, L. Pereira, Prioritising crop wild relatives to enhance agricultural resilience in sub‐Saharan Africa under climate change, Plants People Planet, 4 (3) (2022) 269 - 282, https://doi.org/10.1002/ppp3.10247

[42] M. Shamsudduha, R.G. Taylor, M.I. Haq, S. Nowreen, A. Zahid, K.M.U. Ahmed, The Bengal Water Machine: Quantified freshwater capture in Bangladesh, Science, 377 (6612) (2022) 1315 - 1319, https://doi.org/10.1126/science.abm4730

[43] T.S. Sida, F. Baudron, H. Kim, K.E. Giller, Climate-smart agroforestry: *Faidherbia albida* trees buffer wheat against climatic extremes in the Central Rift Valley of Ethiopia, Agric. For. Meteorol., 248 (2018) 339 - 347, https://doi.org/10.1016/j.agrformet.2017.10.013

[44] R.J. Singh, J.S. Deshwal, N.K. Sharma, B.N. Ghosh, R. Bhattacharyya, Effects of conservation tillage based agro-geo-textiles on resource conservation in sloping croplands of Indian Himalayan Region, Soil Tillage Res., 191 (2019) 37 - 47, https://doi.org/10.1016/j.still.2019.03.012

[45] G.N. Swamy, A.V. Nagavani, Y.R. Ramu, K.C. Nataraj, M. Sadhineni, Effect of integrated nutrient management on nitrogen, phosphorus & potassium uptake and productivity of groundnut (*Arachis hypogaea* L.) under rainfed and protective irrigated condition, Int. J. Chem. Stud., 7 (3) (2019) 4270 - 4274, https://www.chemijournal.com/archives/2019/vol7issue3/PartBR/7-3-477-211.pdf

[46] T. Thorlakson, H. Neufeldt, Reducing subsistence farmers’ vulnerability to climate change: evaluating the potential contributions of agroforestry in western Kenya, Agric. Food Secur., 1 (1) (2012) 1 - 13, https://doi.org/10.1186/2048-7010-1-15

[47] P.H. Vaidya, A.S. Dhawan, Degraded land hybridization with tank silt: Impact on soil quality and productivity of soybean, Indian J. Dryland Agric. Res. Dev., 30 (2) (2015) 30 - 36, https://doi.org/10.5958/2231-6701.2015.00022.6

[48] S.S. Wadne, P.H. Vaidya, A.S. Shrivastav, D.A. Sarda, Evaluation of tank silt hybridized soil and its impact on yield of soybean and pigeon pea in Latur District Maharashtra, Int. J. Chem. Stud., 8 (3) (2020) 2965 - 2970, https://doi.org/10.22271/chemi.2020.v8.i3aq.9662

[49] B. Wang, H. Yan, Z. Xue, G. Liu, Nature-Based Solutions Benefit the Economic–Ecological Coordination of Pastoral Areas: An Outstanding Herdsman’s Experience in Xilin Gol, China, Land, 11 (1) (2022) 107, https://doi.org/10.3390/land11010107

[50] H. Zhang, W. Chen, B. Zhao, L.A. Phillips, Y. Zhou, D.R. Lapen, J. Liu, Sandy soils amended with bentonite induced changes in soil microbiota and fungistasis in maize fields, Appl. Soil Ecol., 146 (2020) 103378, https://doi.org/10.1016/j.apsoil.2019.103378

[51] Y. Zhu, H. Chen, J. Fan, Y. Wang, Y. Li, J. Chen, J. Fan, S. Yang, L. Hu, H. Leung, Genetic diversity and disease control in rice, Nature, 406 (6797) (2000) 718 - 722, https://doi.org/10.1038/35021046

[52] K.A. Garrett, K.F. Andersen, F. Asche, R.L. Bowden, G.A. Forbes, P.A. Kulakow, B. Zhou, Resistance Genes in Global Crop Breeding Networks, Phytopathology, 107 (10) (2017) 1268 - 1278, https://doi.org/10.1094/PHYTO-03-17-0082-FI

[53] C. Li, E. Hoffland, T.W. Kuyper, Y. Yu, C. Zhang, H. Li, F. Zhang, W. van der Werf, Syndromes of production in intercropping impact yield gains, Nat. Plants, 6 (6) (2020) 653-660, https://doi.org/10.1038/s41477-020-0680-9

[54] M.M. Mezeli, S. Page, T.S. George, R. Neilson, A. Mead, M.S.A. Blackwell, P.M. Haygarth, Using a meta-analysis approach to understand complexity in soil biodiversity and phosphorus acquisition in plants, Soil Biol. Biochem., 142 (2020) 107695, https://doi.org/10.1016/j.soilbio.2019.107695

[55] C. Tonitto, M.B. David, L.E. Drinkwater, Replacing bare fallows with cover crops in fertilizer-intensive cropping systems: A meta-analysis of crop yield and N dynamics, Agric. Ecosyst. Environ., 112 (1) (2006) 58 - 72, https://doi.org/10.1016/j.agee.2005.07.003

[56] A. van der Schatte Olivier, L. Jones, L. Le Vay, M. Christie, J. Wilson, S.K. Malham, A global review of the ecosystem services provided by bivalve aquaculture, Rev. Aquac., 12 (1) (2020) 3 - 25, http:s://doi.org/10.1111/raq.12301
